# Empathic Conversational Agent Platform Designs and Their Evaluation in the Context of Mental Health: Systematic Review

**DOI:** 10.2196/58974

**Published:** 2024-09-09

**Authors:** Ruvini Sanjeewa, Ravi Iyer, Pragalathan Apputhurai, Nilmini Wickramasinghe, Denny Meyer

**Affiliations:** 1 School of Health Sciences Swinburne University of Technology Hawthorn Australia; 2 School of Computing, Engineering and Mathematical Sciences La Trobe University Bundoora Australia

**Keywords:** conversational agents, chatbots, virtual assistants, empathy, emotionally aware, mental health, mental well-being

## Abstract

**Background:**

The demand for mental health (MH) services in the community continues to exceed supply. At the same time, technological developments make the use of artificial intelligence–empowered conversational agents (CAs) a real possibility to help fill this gap.

**Objective:**

The objective of this review was to identify existing empathic CA design architectures within the MH care sector and to assess their technical performance in detecting and responding to user emotions in terms of classification accuracy. In addition, the approaches used to evaluate empathic CAs within the MH care sector in terms of their acceptability to users were considered. Finally, this review aimed to identify limitations and future directions for empathic CAs in MH care.

**Methods:**

A systematic literature search was conducted across 6 academic databases to identify journal articles and conference proceedings using search terms covering 3 topics: “conversational agents,” “mental health,” and “empathy.” Only studies discussing CA interventions for the MH care domain were eligible for this review, with both textual and vocal characteristics considered as possible data inputs. Quality was assessed using appropriate risk of bias and quality tools.

**Results:**

A total of 19 articles met all inclusion criteria. Most (12/19, 63%) of these empathic CA designs in MH care were machine learning (ML) based, with 26% (5/19) hybrid engines and 11% (2/19) rule-based systems. Among the ML-based CAs, 47% (9/19) used neural networks, with transformer-based architectures being well represented (7/19, 37%). The remaining 16% (3/19) of the ML models were unspecified. Technical assessments of these CAs focused on response accuracies and their ability to recognize, predict, and classify user emotions. While single-engine CAs demonstrated good accuracy, the hybrid engines achieved higher accuracy and provided more nuanced responses. Of the 19 studies, human evaluations were conducted in 16 (84%), with only 5 (26%) focusing directly on the CA’s empathic features. All these papers used self-reports for measuring empathy, including single or multiple (scale) ratings or qualitative feedback from in-depth interviews. Only 1 (5%) paper included evaluations by both CA users and experts, adding more value to the process.

**Conclusions:**

The integration of CA design and its evaluation is crucial to produce empathic CAs. Future studies should focus on using a clear definition of empathy and standardized scales for empathy measurement, ideally including expert assessment. In addition, the diversity in measures used for technical assessment and evaluation poses a challenge for comparing CA performances, which future research should also address. However, CAs with good technical and empathic performance are already available to users of MH care services, showing promise for new applications, such as helpline services.

## Introduction

### Background

An escalation in mental health (MH) diagnoses in the community, inadequate facilities, and a MH care workforce that does not meet demand are placing extraordinary pressures on an already strained system [1]. This service gap creates a significant opportunity for MH care interventions, enhanced using recent advances in modern technologies. Conversational agent (CA) platforms using artificial intelligence (AI) via machine learning (ML) techniques have emerged within the MH care domain, providing additional functionalities and support to address this gap [2]. Examples of CAs that use ML include Woebot, providing cognitive behavioral therapy [3]; Wysa, providing MH support by checking depressive symptoms [4]; Saarthi, trained to provide personalized and empathic support to patients via therapeutic techniques [5]; and Empathetic Research IoT Network, a chatbot that provides access to MH resources for students in need [6]. However, the lack of acceptance of CAs in the MH domain remains a barrier to the uptake of these innovations, and the lack of empathy often displayed by CAs contributes to end-user mistrust [7].

Empathy in patient care has been defined by the World Health Organization as an understanding of the patient’s experiences, concerns, and perspectives, combined with a capacity to communicate this understanding and an intention to help [8]. Counselor empathy is an essential feature that enhances therapeutic outcomes for patients and can be measured via therapeutic alliance [9,10]. The same is true for CA-human interactions, where empathy exhibited by a CA system helps build rapport, encouraging users to more frequently engage with the CA system [11]. Contextual awareness, which allows CAs to respond to a user’s current emotional situation when suggesting appropriate interventions, also facilitates empathic CA communication [12]. Both trustworthiness of the CA (as perceived by the user) and contextual awareness of the user’s situation (as detected by the CA) are, therefore, important considerations when building an empathic CA. Empathy serves to enhance the bidirectional interaction between the CA and the end user [13].

Assessment of the effectiveness of CA platforms has received little attention in the MH care sector [14]. For the impact of these systems to be fully realized, these platforms need to meet the requirements of end users, which suggests a key role for lived experience and coproduction. The validity and reliability of these new digital technologies also need to be reviewed by MH care decision-makers and professionals to ensure successful integration in the sector [15]. In addition, evaluations need to assess the ability of such platforms to reduce symptoms of mental illness [16] while also enhancing user well-being and ensuring that patients feel understood [13]. However, any such evaluation needs to be conducted in the context of the role envisaged for the CA, considering the success of the bidirectional interaction described earlier.

While there are existing reviews exploring the efficacy of CAs designed for MH care [10,17,18], to our knowledge, this is the first review to specifically examine how these empathic CAs are designed and evaluated. A comprehensive systematic review and meta-analysis of AI-based CAs for promoting MH was conducted by Li et al [17], with a focus on the intervention and technical characteristics of effective CAs. The effectiveness of the CA designs was captured through user feedback. The meta-analysis explored the role of the CA, AI techniques, and delivery platforms that contributed to the success of these designs. In a similar review, Gaffney et al [18] targeted CA interventions for treating MH problems, with a specific focus on user experience outcomes as measures of efficacy. Another such study explored the evidence of effectiveness with regard to improving symptoms of MH conditions [19]. A critical finding of this review was that empathic response and personalization were significant facilitators of efficacy in these systems. However, the incorporation of this crucial empathy component within CAs has not been studied in any depth within the MH sector. Existing reviews have tended to focus on the inability of CAs to respond to unexpected user inputs rather than their ability to demonstrate empathy [19].

### Objectives

This review aimed to assess the types of CA designs found in the MH care sector that are specifically tailored to convey empathy. It also aimed to describe the methods used to evaluate these empathic designs from a technical and implementation perspective. Therefore, this review considered how empathy has been engineered and the limitations identified with its use by a CA from a human perspective. There were three objectives: (1) to identify existing empathic CA design architectures within the MH care sector and to assess their technical performance in detecting and responding to user emotions appropriately; (2) to describe the approaches used to evaluate empathic CAs within the MH care sector in terms of their acceptability to users; and (3) to identify limitations and future directions for empathic CAs in MH care.

## Methods

### Database Search

A systematic literature search was conducted across 6 academic databases (Web of Science; Scopus; EBSCOhost: Academic Search Complete; CINAHL Complete; Computers and Applied Sciences Complete; and IEEE Xplore) for journal articles and conference proceedings from January 1, 2010, to September 30, 2023. The period of data capture dates from the time when AI-informed CA technology emerged as a distinct area of research [20], and conference proceedings were included to ensure that the most recent studies could be included.

The search terms covered 3 topics: “conversational agents,” “mental health,” and “empathy.” Possible keywords were broadened using synonyms for each topic, pilot searching of existing literature, and discussion among research team members. Boolean operators combined different keywords and their synonyms to establish the final search strategy. Wildcards were included (eg, empath* = empathic). Medical Subject Heading terms were used where appropriate. An example of the search syntax is available in Multimedia Appendix 1 [4-6,21-36].

### Eligibility Criteria

Publications discussing CA interventions for the MH care domain were eligible for the review. There were no restrictions on research design (eg, observational designs and narrative review). This review considered both textual and vocal modes of interaction with the CA. Publications were included if they referred to CA empathy or related terms (eg, emotional intelligence, emotional awareness, and compassion). Publications that did not feature a methodology section that detailed CA design, types of data sets, and participants were excluded. Systematic reviews, scoping reviews, and meta-analyses were excluded. Publications that used data inputs other than text and vocal cues (eg, facial recognition) were also excluded. Multimedia Appendix 1 provides the full-text screening checklist.

### Screening

**Figure 1:**
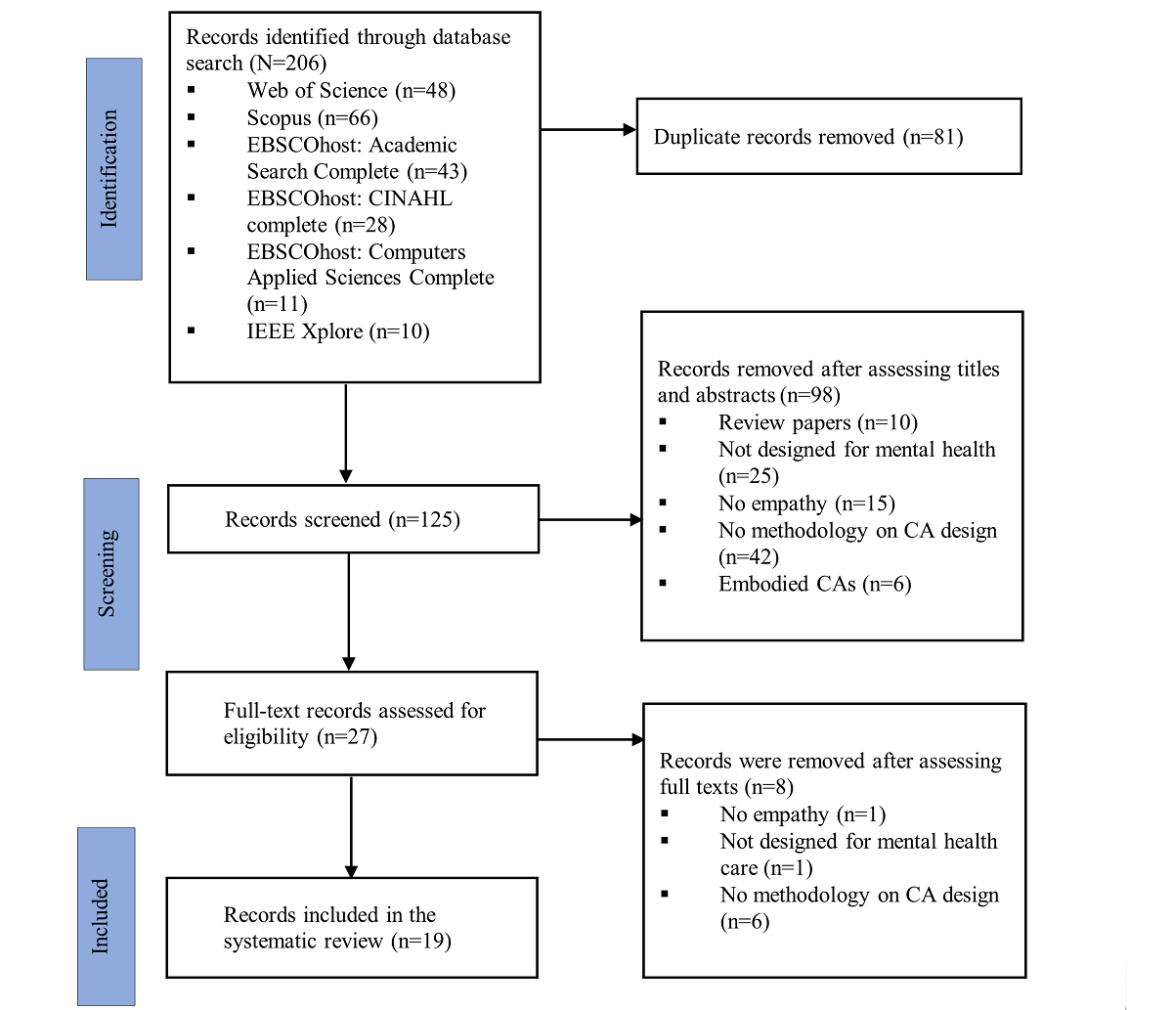
PRISMA (Preferred Reporting Items for Systematic Reviews and Meta-Analyses) procedure applied. CA: conversational agent.

Eligible references were exported to the EndNote (version 20; Clarivate) software [37], where duplicates were removed. The first author (RS) conducted the title and abstract search, mapping against the eligibility criteria. A full-text screening was then performed by the first author and by 2 other authors, DM and RI, independently. Any disagreements on full-text screening were discussed, and an agreement was reached before proceeding. Figure 1 illustrates the PRISMA (Preferred Reporting Items for Systematic Reviews and Meta-Analyses) flowchart describing the screening process. PRISMA checklist is reported in Multimedia Appendix 2.

Data including details on the study designs, how empathy was evaluated, and the types of CA architectures used were extracted to obtain a summary of all findings (Multimedia Appendix 1).

### Quality Assessment

The Joanna Briggs Institute critical appraisal tool was used to assess the methodological quality of the papers shortlisted while also considering the extent to which each study addressed the possibility of bias in design, conduct, and analysis [38]. This appraisal tool was specifically designed for the assessment of the variety of study designs encountered in this systematic review. Decisional criteria were answered with *yes, no, unclear, or not applicable*. The proportion of *yes* responses relative to the total number of assessment questions was used for quality assessment purposes. Separate quality assessments were conducted for publications that included a description of the implementation as well as the design of the CA platform and for publications that included only a description of the design.

### Risk of Bias

Risk of bias was assessed using the revised Cochrane risk-of-bias tool for randomized trials This included risks of bias due to randomization, deviations from the intended intervention, missing data, the measurement of outcomes, and the selection of results. The risk of bias in nonrandomized studies of interventions tool was used to evaluate the nonrandomized studies.

## Results

### Overview

A total of 19 studies met all the inclusion criteria. The study characteristics are summarized in Table 1.

**Table 1:** Study characteristics.

Study	CA^a^	Training database	Aim of the study	Evaluation measures for detecting and responding to user emotions	Mode of exchange	Analysis model for generating empathic responses
Jiang et al [21], 2022	Replika	14 Chinese female users (aged 19-26 years)	Explore types of mediated empathy that occur in human-AI^b^ interactions	In-depth interviews and survey results: user ratings of empathy	Text and voice	Transformer architecture
Brocki et al [22], 2023	Serena	Trained on “Pushshift” Reddit data set and tested on psychotherapy transcript	Help improve outcomes of counseling by lowering barriers to access	Survey results: user ratings of engagement and helpfulness	Text	Transformer architecture
Persons et al [6], 2021	ERIN^c^	15 undergraduate students	Help users with finding resources about sensitive issues	Survey results: user ratings for experience	Text	Rule-based architecture
Trappey et al [23], 2022	Virtual reality empathy-centric counseling CA	120 university students	Provide complementary support for students who were troubled	Survey results: user ratings of stress levels, life impact, and psychological sensitivity	Voice and text	Transformer architecture
Ghandeharioun et al [24], 2019	EMMA^d^	39 participants	Delivery of just-in-time MH^e^ interventions	Survey results: user ratings of preference; behavioral metrics: user engagement	Text	Hybrid architecture
Meng and Dai [25], 2021	AI CA	278 participants from Midwestern University	Check whether the CA’s emotional support was effective in reducing people’s stress and worry	Survey results: user ratings of stress, worry, and perceived support	Text	Transformer architecture
Goel et al [26], 2021	Empathic CA with an attention mechanism	Trained with the Facebook AI Empathic Dialogue data set	Support users express their feelings and anxious thoughts	None	Text	Neural network architecture
Adikari et al [27], 2022	Empathic CA	Data set from Cancer Chat Canada	Provide empathic patient-centered MH care	Behavioral metrics for user engagement	Text	Hybrid architecture
Inkster et al [4], 2018	Wysa	129 users with self-reported symptoms of depression	Evaluation of the effectiveness and engagement levels of Wysa	Survey results for symptom assessment	Text	Unspecified ML^f^ architecture
Beredo and Ong [28], 2022	Vhope	Senior high school and college students (aged 17-20 years)	Help the students maintain their well-being	Response ratings provided by experts	Text	Hybrid architecture
Rathnayaka et al [29], 2022	Bunji	Australian mobile users on Google Play Store	Remote health monitoring	Survey results for symptom and mood assessment	Text	Unspecified ML architecture
Morris et al [30], 2018	Koko	37,169 individuals who signed up for the Koko platform	A corpus-based approach to simulate expressed empathy	Response ratings provided by users	Text	Hybrid architecture
Ghandeharioun et al [31], 2019	A behavioral change CA	39 participants (n=7, 18% were female, and n=32, 82% were male)	Conduct experience sampling	Survey results: user ratings of likability and CA intelligence	Text	Rule-based architecture
Saha et al [32], 2022	Empathic CA	Data set: conversations between the support seekers who were depressed	Generate empathic and motivational responses	Response ratings by users for fluency, adaptability, and motivation	Text	Transformer architecture
Agnihotri et al [33], 2021	Topic-driven and affective CA	Data set: “ScenarioSA” with affective state labels	Tackle the emotional and contextual relevance for mental well-being	Response ratings for emotional relevance	Text	Transformer architecture
Rani et al [5], 2023	Saarthi	None	None	None	Text	Unspecified ML architecture
Alazraki et al [34], 2021	An empathic AI coach	23 participants recruited through crowd working websites	Achieve a high level of engagement during web-based therapy sessions	Survey results: user ratings of empathy and expert ratings of fluency	Text	Hybrid architecture
Gundavarapu et al [35], 2022	A CA companion	Data set: created using sources such as Wikipedia	Provide emotional support, without judgment	None	Text	Neural network architecture
Mishra et al [36], 2023	Counseling CA	A novel conversational data set	Provide MH and legal counseling	Survey results: user ratings of empathy	Text	Transformer architecture

^a^CA: conversational agent.

^b^AI: artificial intelligence.

^c^ERIN: Empathetic Research IoT Network.

^d^EMMA: Emotion-aware mHealth agent.

^e^MH: mental health.

^f^ML: machine learning.

Of the 19 studies, 6 (32%) were conducted in the United States and 6 (32%) in India. In addition, 1 (5%) study each from Australia, Canada, China, the Philippines, Poland, Switzerland, and the United Kingdom were also included. The year of publication is summarized in Multimedia Appendix 3, indicating a sharp rise in the number of publications since 2022. Most studies, 14 (74%) out of 19, described both design and human evaluations. The types of study designs among the 19 studies included are 9 (47%) cross-sectional studies, 5 (26%) randomized controlled trials (RCTs), 4 (21%) quasi-experimental designs, and 1 (5%) qualitative study. Only 5 (26%) of the 19 studies referred to an explicit definition of empathy, as summarized in Textbox 1.

**Definitions of empathy.**

**Studies and definition of empathy**

- Jiang et al [21], 2022
  Empathy processing is a situation-specific, cognitive-affective state or process with the projection of oneself into another’s feelings, actions, and experiences.
- Trappey et al [23], 2022
  Roger’s [39] definition of empathy:
    - Level 1: responding to an individual’s explicitly expressed meaning and feelings with a simple repetition of basic understanding.
    - Level 2: responding to the implicit, half-expressed, or implied feelings of the person with corresponding emotional words to acknowledge them and bring their true feelings to the surface.
    - Level 3: recognizing the individual’s confusing and contradictory feelings that subconsciously obscure what they really care about, capturing the core of the emotion, and then responding to the patient’s desire with affirmations.
    - Level 4: when the person is suppressing their feelings or not expressing their feelings in the conversation, guessing their intentions from what they are describing, capturing the core of the emotion, and responding to it directly or indirectly in a way that is acceptable to the person.
- Rathnayaka et al [29], 2022
  Empathic engagement means, “making the impression of a credible and trustworthy conversation partner that can hear you out and offer a detached point of view on things.”
- Saha et al [32], 2022
  Empathy or empathic interactions refer to the ability to feel the emotions and experiences of others [40].
- Alazraki et al [34], 2021
  Definition of empathy by Barrett-Lennard [41]:
    - First phase: where the listener sympathizes and resonates with what is being expressed by the speaker.
    - Second phase: where the listener compassionately responds to the speaker. Third phase: where the speaker assimilates the listener’s response.

Keywords used to identify a CA varied across studies from “chatbot” (9/19, 47%) to “conversational agent” (6/19, 32%) to “dialog system” (2/19, 11%) to “virtual assistant” (1/19, 5%) to “conversational AI agent” (1/19, 5%). The mode of interaction chosen by most of the CA designs, 17 (89%) out of 19, was text (eg, live chat, symptom checker, and text-based counseling), with voice interactions being used in interactive avatar and counseling roles in 2 (11%) studies.

In the *Technical Design of the CAs* section, we consider the technical designs used for these CAs and their performance in detecting and responding to user emotions before discussing how human-user evaluations were conducted and the conclusions reached from these evaluations.

### Technical Design of the CAs

**Figure 2:**
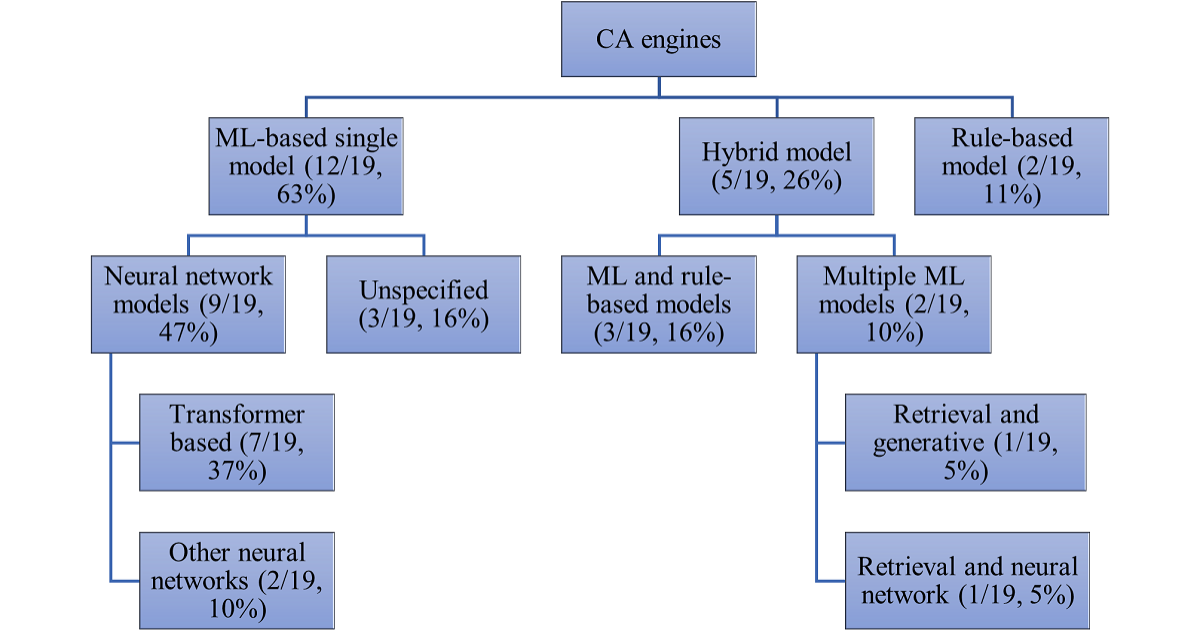
Types of conversational agent (CA) architectures. ML: machine learning.

The types of CA architectures (or engines) considered by the authors included a mix of recent technologies, as summarized in Figure 2, with ML-based architectures used in 12 (63%) out of 19 cases. The transformer-based engine, which learns meaning from context, was used in 7 of the 19 (37%) studies, sometimes in the form of a large language model (LLM). A minority of the papers, 3 (16%) out of 19, did not specify the type of engine used within the design. Hybrid or ensemble models use several models in parallel to improve the accuracy of the overall CA design. A more detailed breakdown of the CA engine types with explanations is shown in Multimedia Appendix 4. Figures S1 and S2 in Multimedia Appendix 4 also illustrate how a single engine and a hybrid engine work with user input to provide an empathic response.

Transformer-based engines included Bidirectional Encoder Representations Transformer (BERT), Sentence-BERT, Robustly Optimized-BERT, Generative Pre-trained Transformer 2, and sequence-2-sequence models. Other neural network architecture–based CA designs were incorporated in 2 (11%) of the 19 papers [26,35].

Of the 19 publications, 5 (26%) considered hybrid models. Of these hybrid models, 2 applied a ML model to capture user emotion and then applied a rule-based algorithm to generate appropriate responses in dialogue management [24-27]. For example, EMMA gathered mobile sensor data to infer user mood and then assigned users to appropriate wellness interventions [24]. Once assigned, the CA then responded with emotionally expressive responses selected at random from a set of prescripted phrases using a rule-based approach [27]. In another example, VHope, an internet-based therapist, used a hybrid model containing a retrieval model that deciphered user input combined with a generative model to elicit empathic responses [28].

Among the 19 papers, the 3 (16%) papers using unspecified architectures commenced with natural language processing (NLP) before using various ML approaches. In one example, continuous emotional support via remote MH care monitoring and personalized assistance was provided [29]. MH monitoring was performed by scheduling activities that were meaningful to each user, sending out reminders as encouragement, and forwarding satisfaction surveys to receive feedback.

Overall, 2 (11%) of the 19 publications implemented CA design approaches based on rule-based NLP architectures. For example, a mobile phone–based CA measured the level of emotion in user input and then selected an appropriate empathic response from a set of predefined scripts using a rule-based decision tree [31]. In the *Summary of the Results of the Assessment of the Technical Design of CAs in Terms of Classification Accuracy* section, we will discuss the technical performance of the CAs reviewed.

### Summary of the Results of the Assessment of the Technical Design of CAs in Terms of Classification Accuracy

The accuracy of the designs in detecting and responding to user emotions appropriately is summarized in Table 2. Technical evaluations of the CA designs usually involved comparisons with a “gold standard,” using data not previously used for training the CA.

**Table 2:** Measures used for evaluating the technical performance of CA^a^ designs.

Type of CA assessment	Assessment of user emotions or CA responses	Accuracy measure
Classification of sentiment and issues	User emotions	Mathews correlation coefficient=0.857 [23]
Classification of valence and arousal	User emotions	Accuracy of valence=80.4% [24] Accuracy of arousal=50.4% [24]
Classification of recommended resources (for patients)	User emotions	F1-score=0.87 [27]
Classification of objections during conversations	User emotions	Accuracy=99.2% [4] Specificity=99.7% [4] Precision=74.7% [4] Recall=62.1% [4]
Performance of the topic classifier	User emotions	Accuracy=95% [33] Precision=0.954 [33] Recall=0.947 [33] F1-score=0.95 [33]
Classification for empathy function	User emotions	Accuracy=80.18% [34] F1-score=80.66% [34] W-ACC^b^=0.977 [36] Macro F1-score=0.972 [36]
Prediction of valence and arousal	User emotions	Accuracy of valence=82.2% [24] Accuracy of arousal=65.7% [24]
Accuracy of the response generation	CA responses	BLEU^c^ score=0.126 [26] BLEU-1 score (focused on a single word)=0.161 [32] Perplexity score=50.90 [32] ROUGE-L^d^ score=0.124 [32] Embedding-based metrics: Average=0.733 [32] Extrema=0.377 [32] Greedy=0.478 [32]
Emotion prediction	User emotions	Accuracy Correctly predict the next emotion as positive or negative=79% [27] Proportion of correct emotion out of all emotions predicted=63% [27]
Performance of the language model	CA responses	Perplexity score=9.977 [28] Perplexity score=1.91 [23] Response length=18.71 [23]
Emotion recognition	User emotions	Accuracy=94.96% [34] F1-score=95.10% [34]

^a^CA: conversational agent.

^b^W-ACC: weighted accuracy.

^c^BLEU: Bilingual Evaluation Understudy.

^d^ROUGE-L: Recall Oriented Understudy for Gisting Evaluation–Longest Common Sequence.

A technical evaluation of empathic CA performance was conducted in 17 (89%) of the 19 papers reviewed; however, only 10 (53%) papers reported these results. These studies conducted comprehensive assessments where technical performance was measured in terms of recognition, classification, prediction, and response generation abilities during interactions with end users. The assessments were centered around the ability of the CA to discern user emotions correctly and to respond appropriately. Of the 19 papers, 4 (21%) focused on the CA responses during the technical assessments, while the rest of the studies (n=15, 79%) considered user emotions. A variety of measures were used for each such assessment, highlighting the diversity in evaluation methodologies across studies. These metrics are categorized in detail under the type of CA performance in Multimedia Appendix 5 [4-6,23-34,36].

In general, the performances of the CA designs were satisfactory. The highest classification accuracy for user emotions was reported by ML-based CAs. In one of these studies, a Robustly Optimized-BERT transformer model, which was built integrating 3 classifiers for politeness, counseling strategy, and empathic feedback, achieved good results overall. This empathy classifier achieved excellent performance with a weighted accuracy score of 0.977 and an *F*_1_-score of 0.972 [36]. In a second study, a topic-driven classification model used a Generative Pre-trained Transformer 2 model for generating controlled responses, and the classification model accomplished relatively high scores of accuracy (95%), precision (0.954), and recall (0.947) and an *F*_1_-score of 0.95 [33].

However, high accuracy and a more nuanced response generation were consistently apparent in all the CAs using hybrid architectures [24,27,28,30,34], suggesting that hybrid models lead to enhanced performance in tasks requiring complex understanding of user emotions and the generation of contextual responses.

### Human Evaluation of CAs

Most of the reviewed studies, 16 (84%) out of 19, conducted a human evaluation of the implemented CA designs. Acceptability by end users was evaluated in terms of user experience, satisfaction, and levels of engagement. A detailed summary of the human evaluations of these designs is presented in Multimedia Appendix 5.

The human evaluation was performed by only CA users in most cases (13/16, 81%), while experts in the field of MH contributed to the process of assessing the CA in the remaining studies (3/16, 19%). Table 3 summarizes the empathy measures used in these papers.

**Table 3:** Measurement of empathy in CAs^a^.

Study and year	The method of empathy measurement	How was empathy measured?	Who did the evaluation?	Evaluation results
Jiang et al [21], 2022	Self-reports: In-depth interview responses Multiple response ratings	Using the RoPE^b^ scale (binary responses) and QCAE^c^	Replika users provided the empathy ratings	Perceived cognitive empathy was higher than perceived affective empathy
Beredo and Ong [28], 2022	Self-reports: Response ratings	Affect criterion or empathy was measured using a binary scale of 0 (no) to 1 (yes)	Evaluated by 3 experts who studied and practiced psychology	Responses were rated 79% empathic
Alazraki et al [34], 2021	Self-reports: Multiple response ratings	Multiple ratings to evaluate the perceived level of empathy, with ratings ranging from strongly disagree to strongly agree on a 5-point Likert scale	Evaluated by users 2 separate clinicians specialized in MH^d^ also evaluated the chatbot personas	When interacting with the Kai persona, 75% of users agreed that the bot was empathic Interaction with other study personas achieved a 56% empathic rating
Mishra et al [36], 2023	Self-reports: Response ratings	A single 5-point Likert scale	6 evaluators rated each dialogue interaction for empathy Empathy ratings by evaluators cross-validated for quality by government-run institutions	Average empathy rating=57%
Agnihotri et al [33], 2021	Self-reports: Response ratings	Emotional relevance is rated using a single 5-point Likert scale	Evaluated by 3 human annotators—male nonnative English speakers from a technical university with an average age of 21 years	When an empathic response generator was used, emotional relevance=61.4% When a topic classifier was added, emotional relevance=43%

^a^CA: conversational agent.

^b^RoPE: Robot’s Perceived Empathy.

^c^QCAE: Questionnaire of Cognitive and Affective Empathy.

^d^MH: mental health.

Alazraki et al [34] conducted a cross-sectional study with 23 volunteers and 2 clinicians who engaged with a web-based chatbot platform using 4 prescripted conversations of different CA personas. An anonymous web-based questionnaire collected participant feedback regarding the level of empathy displayed by the chatbot, engagement levels, and the ability of the chatbot to identify emotions in the participant. The survey results revealed that 75% of users agreed that the CA persona Kai was empathic, 63% found it engaging, and 75% rated it as useful. In contrast, Beredo and Ong [28] asked 3 psychologists to provide feedback on chatbot user logs. Empathy was measured using the affect criterion, a measure of the ability of the CA to read and respond to users with empathy, along with performance and humanlike characteristics. On the basis of expert feedback, 67% of the CA responses were relevant, 78% seemed human, and 70% were empathic.

In an RCT, a group of 39 participants were randomly allocated to a treatment group interacting with the emotion-aware chatbot EMMA, while a control group (n=39) was assigned to an emotionally nonexpressive chatbot, with 2 weeks of monitoring in each case [24]. The participants engaging with EMMA showed higher frequency of interactions and responded quicker than the control group. The feedback of the users was useful in understanding how empathy was perceived during the study.

The only qualitative experimental study involved an AI-based chatbot, Replika, designed to improve resilience and user well-being [21]. The author followed an ethnographic approach for their study of empathy, asking users to download the Replika application and write down reflective notes on their conversations with Replika. The results of this study expand the empathy theories within human conversations to human-AI interactions through variations in cognitive empathy, affective empathy, and empathic responses. A list of technical terms used in the paper is further explained in Multimedia Appendix 6.

### Risk of Bias and Quality Assessment Results

The included RCTs showed a low risk of bias on the revised Cochrane risk-of-bias tool. Of the 14 nonrandomized studies included in the review, all showed a moderate to high risk of bias. A total of 5 (36%) studies [27,32-34,36] were moderately biased, and 1 (7%) study [28] was seriously biased according to the risk of bias in nonrandomized studies of interventions tool. The Joanna Briggs Institute quality assessment results were generally low when only the design component of the studies was assessed, with 32% (6/19) of the papers receiving a score of 0. However, an overall moderate quality was seen in publications when both the design and implementation stages were appraised. Multimedia Appendix 7 [4-6,21-36] shows the quality assessment results.

## Discussion

### Principal Findings

The study and use of CA technology have been the subject of extensive research across many fields, such as education, customer service, and health care. Moreover, there are reviews focusing on AI-based CAs, their effectiveness, and their impact in the realm of MH care [17,18,42]. While these reviews offer significant insights into AI-based CA designs in MH care, the importance of empathy is not central. Although these reviews suggest the need for empathy in CA innovations in MH care, they do not consider CA designs specifically aimed at generating and evaluating empathy. To address this gap, this review compares various empathic CA designs, their effectiveness in detecting and responding to user emotions, and their acceptability to users.

### CA Designs

This review has found that most researchers used an ML-based transformer engine for designing empathic CAs, achieving excellent classification and prediction results. Surprisingly, several researchers used rule-based architectures and retrieval engines. While lacking the sophistication of transformer-based engines in terms of comprehension, rule-based approaches were able to efficiently identify keywords and themes, ensuring that consumer needs were addressed within a limited number of categories. Rule-based systems are comparatively easy to design and implement, allowing for a trade-off between classification accuracy and economic feasibility. However, rule-based systems tend to generate more predictable, inflexible, and repetitive responses compared to advanced LLM engines and, therefore, might be more suitable for providing simple information to managers and MH care workers, rather than responding to end users requiring more nuanced responses.

Hybrid architecture seems best suited to the detection of user emotion followed by the retrieval of a suitable response. Therefore, having >1 model appears to facilitate a more robust model output. This is supported by the superior accuracies achieved by hybrid architectures in the classification and prediction tasks. The hybrid model of Adikari et al [27] achieved the highest accuracy of 87% (*F*_1_-score=0.87) in recommending a resource based on the concerns expressed by the patients. However, the highest accuracy in emotion recognition (95% accuracy in identifying sadness, anger, fear, and happiness) was obtained by Alazraki et al [34]. The combined features of high accuracy and improved user experience probably make these the best performing CAs within the review.

While the use of such robust LLMs has significantly improved language-based CA technology, it is important to recognize that these models are not without disadvantages [43]. These models have been found to perpetuate biases with regard to gender, race, and MH conditions present in the training data [44,45]. Such biases can strengthen gender stereotypes and reduce response accuracy when dealing with users from diverse cultural backgrounds, potentially causing harm to users. Such issues may have serious impacts on user trust, the credibility of the empathic CA, and user well-being. Such biases can be mitigated by ensuring that the training data sets represent diverse gender categories, races, and cultural backgrounds and that advanced technical approaches are used to detect and minimize any such biases in the training data [46-48].

Ethical and privacy concerns associated with these LLMs are critical [49,50]. Following ethical guidelines centered around transparency, accountability, and adherence are pivotal to user privacy, while measures to maintain data security through strict access controls and regular security checks also need to be in place. Privacy should be a core component of CA designs, with limitations placed on personal data collection whenever possible [7]. These strategies are especially important for an empathic CA design dealing with users seeking MH care. Any breaches of privacy and ethical guidelines pose a high risk to user mental well-being as well as users’ trust in and acceptance of these new technologies [51]. The AI safety guidelines established by the European Union provide a key foundation for the creation of secure and ethical experiences for users [50].

Due to the complexity of LLMs and the many parameters involved, some models can have high latency in response time, which can cause potential challenges for a real-time CA dealing with vulnerable users waiting for a response. However, the use of parallel processing, optimization techniques, and hardware that supports the requirements of these AI models has facilitated a decrease in execution times [52].

### Human Evaluations of CAs

Among the reviewed publications, human evaluation of chatbots was common. However, only 26% (5/19) of the studies used an RCT design to assess the CA platform. Random assignment to the treatment arm is known to reduce bias while improving the reliability of the experimental results. Any confounding factors are, therefore, likely to be controlled for in an RCT, making it important to overcome the practical difficulties these designs present in this context. RCTs provide the opportunity to observe user experiences with the CA designs over time. Ideally, future studies should consider RCT designs for their human evaluations, and ideally, the long-term effects of the CA can be examined over an extended timeline.

Previous experiences with CAs could be an important confounding factor. On the basis of these experiences, expectations of users regarding CA performance may affect actual engagement with the CA. Previous bad experiences may make it less likely that a user will try to engage fully with a CA, resulting in a less favorable evaluation and satisfaction levels [53]. Another confounding factor could be the rate at which the user likes to communicate. If the CA cannot automatically adapt its speed of response to that preferred by the user, it is likely that this will also impact evaluation results [54].

The human evaluations of CAs in this review focused on their ability to portray empathy, satisfy user needs, provide useful and contextually informed responses, and facilitate user engagement. Most CAs were evaluated as satisfactory by end users. However, among the 19 papers reviewed, only 5 (26%) papers provided quantitative evaluations of CA empathy, and only 5 (26%) papers provided a definition of empathy.

Because empathy has been defined in numerous ways in the literature, it is important that in future studies users are given a framework that guides their perceptions of empathy. Future research on empathic CA designs would, therefore, benefit from a clear and well-established definition of empathy, such as that provided by the World Health Organization [8]. Ideally, standardized scales for perceived empathy should be used to enhance the reliability, comparability, and validity of survey results. In this review, other self-report measures were used as surrogates for empathy, with considerable variation in the types of scales used. However, self-report scales are subjective and prone to bias, with different meanings based on users’ lived experiences [55]. Ideally, the impact of the CA on MH outcomes should also be assessed. Only 2 (11%) of the 19 papers in this review [4,29] used the Patient Health Questionnaire as their measure of MH outcomes, while 2 (11%) other papers considered stress levels in their evaluation [23,25].

Furthermore, the human evaluations were mostly conducted by study participants. Experts and professionals in the field of MH care were rarely consulted. There is a need for greater consultation with focus groups and user groups to ensure that the CA design best reflects the needs of all stakeholders [22]. Future research in this area should also consider an iterative design framework, incorporating the co-design and coevaluation of prototypes involving all stakeholders [22].

In summary, there were deficiencies in all the human evaluations included in this review. Only 5 (26%) of the 19 papers in this review included a direct evaluation of CA empathy in the design, while the rest (n=14, 74%) were more concerned with general user satisfaction. Only 2 (40%) of 5 these studies used multiple rating scales to measure the level of empathy portrayed by a CA, and only 1 (20%) of 5 these studies [34] considered evaluations by both users and clinicians. However, there were 4 studies that did consider the impact of the CA on MH outcomes.

### Future Opportunities

A significant limitation of the CAs reviewed was the use of only textual input in all but 2 (11%) of the 19 studies where voice data were included, thus losing a valuable opportunity to leverage alternative and powerful forms of data input for evaluating empathy.. A range of vocal characteristics have been associated with the detection of suicide risk and psychological distress, which suggests that vocal characteristics might provide a natural extension for the detection of levels of empathy [56,57]. The omission of voice data is surprising given that empathy is communicated predominately through vocal cues. However, textual information is not without its advantages. As we have shown in this review, NLP approaches have been used to successfully detect and convey empathy by CAs. A novel approach would be to leverage both streams of information to identify vocal characteristics indicative of different levels of empathy in addition to textual cues. Characteristics of vocal and textual cues that are associated with empathy could be combined to create a CA design to attend to users of MH care facilities such as helpline services, patient triage, and emergency services [21,23].

Creating a CA design that accurately portrays empathy and adjusts the level of empathy to match the emotional status of patients is a significant challenge. Effective vocal interaction often faces hurdles due to technical issues in voice analysis, including the smooth processing and interpretation of data. These challenges are compounded by poor audio quality [58]; the presence of overlapping psychological states in users; and linguistic variability influenced by culture, age, gender, and accents [59-61]. The use of high-quality audio devices to capture user voice [62, 63] and the use of training data sets reflecting diverse human demographic features are two challenges in algorithm development aiming to provide effective vocal interaction in CAs in real time.

The integration of an empathic CA with voice analysis capabilities into crisis helpline services could benefit users and the service provider. Attending to callers during peak hours for the collection of demographic information, triage, and risk assessment of callers using their voice patterns are some of the possible roles that CAs could fulfill. The involvement of CAs in these capacities could help reduce caller wait times, streamline processes, and ensure 24-hour service availability while providing a nonjudgmental and sensitive interaction for users within a safe environment. Improved empathy portrayal by the CA would help enhance user engagement and CA acceptability, helping reduce the gap between the demand and supply of available crisis helpline services.

### Summary

This review confirms that empathy is an important characteristic for CA implementation for MH care. It highlights the strengths of the ML-based architectures when it comes to CA design and provides evidence of both technical and human assessments of CA performance. The need for improvement in measures used for detecting the level of empathy exhibited by CAs is manifest. The importance of AI safety regarding ethical and privacy concerns is a neglected area and should be considered as a priority for future designs. The promise of empathic CA applications that use vocal inputs and outputs is another area warranting further research, with opportunities for crisis helpline services.

### Limitations of the Review

The studies included in this review presented a mix of methods, which made it challenging to compare and analyze the results. This relates to the diversity in the CA designs included, along with the different data formats obtained through human evaluations, such as survey results, response ratings, and interview feedback. The methods used to assess the accuracy of the technical designs were also varied, and a lack of empathy definitions and standard measures for perceived empathy made study comparisons difficult.

The quality rating of the studies emphasized the need for the complete reporting of CA designs as well as rigorous evaluation. Deficiencies in these areas meant that the quality ratings for several papers were low. Evaluation guidelines were often missing, which made it challenging to appraise the performance of these systems. Classification accuracy and the accuracy of the responses generated were assessed using a variety of methods, further complicating this comparison.

### Conclusions

The objective of this systematic review was to identify the existing architectures of empathic CA designs and the types of CA design assessments used in MH care. A further aim was to determine how CA empathy is evaluated and to examine the limitations and future ideas for CAs in this specific context. More than half of the selected papers used the latest technologies in CA architectures, including designs developed using ML-based transformer engines (eg, LLMs). Evaluations of technical capabilities were conducted in most of the papers and demonstrated good levels of accuracy.

This review suggests that a hybrid design is ideally used for the design of an empathic CA, allowing an initial assessment of user emotion before any CA response is developed. This review indicates that human feedback is required to assess the extent to which the CA is successful in demonstrating empathy. It is recommended that well-validated scales be used for this purpose. Further research on the portrayal of empathy in CAs for MH care would benefit by involving cocreation activities, explicit definitions of empathy, and effective evaluation of empathy using standardized empathy scales, as well as by using vocal features associated with empathy in addition to textual cues.

Despite its limitations, this review demonstrates that it is possible to design AI-empowered CAs that evoke empathy within MH care applications, with many of these CAs being rated as satisfactory by human users. This suggests that such CAs could prove beneficial in a range of settings, such as crisis helpline services, gathering data on user characteristics and emotions, and in postvention follow-up, helping to bridge the gap between the existing supply and demand for MH services.

## Appendix

Multimedia Appendix 1 Screening process and study characteristics.

Multimedia Appendix 2 PRISMA (Preferred Reporting Items for Systematic Reviews and Meta-Analyses) checklist 2020.

Multimedia Appendix 3 Evolution of conversational agent (year by year).

Multimedia Appendix 4 Detailed summary of conversational agent types.

Multimedia Appendix 5 Results of conversational agent evaluations.

Multimedia Appendix 6 Dictionary of technical terms.

Multimedia Appendix 7 Risk of bias and quality assessment.

## Abbreviations

AI: artificial intelligence
BERT: Bidirectional Encoder Representations Transformer
CA: conversational agent
LLM: large language model
MH: mental health
ML: machine learning
NLP: natural language processing
PRISMA: Preferred Reporting Items for Systematic Reviews and Meta-Analyses
RCT: randomized controlled trial
