# Supplementary material for: Empathic Conversational Agent Platform Designs and Their Evaluation in the Context of Mental Health: Systematic Review
Source: JMIR Ment Health. 2024 Sep 9;11:e58974. doi: 10.2196/58974 (PMC11420590; doi:10.2196/58974)
Supplement: Multimedia Appendix 1 [file mental_v11i1e58974_app1.docx]

(TITLE-ABS-KEY (chatbot* OR "chat bot*" OR "chat agent*" OR chatterbot* OR voicebot* OR "voice bot*" OR "voice agent*" OR "virtual assistant*" OR "conversational agent*" OR "conversational artificial intelligence" OR "conversational user interface*" OR "conversational bot*" ) )

AND

(TITLE-ABS-KEY (empathy OR empath* OR sympath* OR sympathy OR compassion* OR "emotional intelligence" OR "emotionally aware" OR "therapeutic cue*" OR "therapeutic alliance*" ) )

AND

(TITLE-ABS-KEY ("mental health" OR "mental well being" OR "mental wellbeing" OR distress) )

AND

PUBYEAR > 2009 AND PUBYEAR < 2024 AND (LIMIT-TO ( DOCTYPE , "cp" ) OR LIMIT-TO ( DOCTYPE , "ar" ) ) AND ( LIMIT-TO ( LANGUAGE , "English" ) )

Table S1: Search syntax for the article search

Table S2: Full-Text Screening Criteria

| Inclusion criteria for article screening titles, abstracts and full texts:   1. CAs designed for the mental health care sector. 2. CA designs using voice or text for interactions with the user. 3. Empathic features included in the CA design. 4. Papers including a methodology on CA design. 5. Limited to journal articles and conference papers. 6. Articles published within the years from 2010 to 2023 in the English language.   Exclusion criteria:   1. Systematic review, Scoping review or Meta-analysis papers. 2. Embodied CAs or avatars. |
| --- |

Table S3: Study Characteristics

| **Author** | **Population(P)/Dataset** | **Intervention(I)** | **Comparison(C)** | **Outcome(O)** | **Study Design(S)** | **Empathy recognition**  **by the CA** | **How empathy is defined** | **The measure of empathy** | **How was empathy measured? Who did the evaluation?** | **Mode of interaction** | **Type of CA Framework** |
| --- | --- | --- | --- | --- | --- | --- | --- | --- | --- | --- | --- |
| Q. Jiang et al. | *Chinese women using Replika, A sample of 14 voluntary young Chinese female users aged between 19 and 26 was used. *Their Name, age, gender, education, and relationship status were recorded. Data collected via, *Authors' reflective notes *Text retrieval from online posts about Replika *Participant's observations shared through online discussions *In-depth interviews with recruited users *Ethnography- Qualitative research on a cultural group. | Exploring the possibility and types of mediated empathy in human-AI interactions using chatbot, Replika . | None | Identified five types of mediated empathy with varying degrees of cognitive empathy, affective empathy, and empathic response involved in human-AI interaction through this qualitative study. | Qualitative study | None | Empathy processing is a situation-specific, cognitive-affective state or process with the projection of oneself into another’s feelings, actions, and experiences. | *In-depth interview responses - User perceptions of empathy | A questionnaire and a scale *The Robot’s Perceived Empathy (RoPE) Scale-Binary responses *Questionnaire of Cognitive and Affective Empathy (QCAE) * A theoretical framework of empathy was used. *Replika users provided the empathy ratings | Voice and text | Transformer engine (GPT-3)-A theoretical framework |
| L. Brocki et al. | *Postprocessing algorithms detect contradictions, improve coherency, and remove repetitive answers. *Model pretrained on Pushshift Reddit Dataset with 651 million submissions and 5.6 billion comments. *Fine-tuned the model on transcripts of counselling and psychotherapy transcripts. -14,300 patient’s prompt and counsellor’s answer pairs; Fine-tuned using ParlAI platform | A deep learning (DL) dialogue system called Serena for counselling to help improve outcomes by lowering barriers to access. | None | The dialogue system proved the potential to provide a low-cost and effective complement to traditional human counsellors with fewer barriers to access. | Cross-sectional | None | None | None | NO EMPATHY *Survey results *Users are asked to rate the degree to which the model understands their messages and whether they find the generated responses engaging and helpful. - In a questionnaire. | Text | *Seq2seq transformer-based generative model- 2.7 billion parameter,2 encoder layers, 24 decoder layers, 2560 dimensional embeddings,32 attention heads. |
| B. Persons et al. | *Interviews with key informants (Co-design) *User interviews to verify needs user needs and preferences. *Prototype implementations | User experience-driven innovation chatbot (UXDI)framework, ERIN to help with finding resources about sensitive issues | Participants were randomly assigned to two groups: laptop and mobile. | The preliminary results showed that the user experience of the chatbot was almost significantly better in the mobile group and people in that group were almost significantly more likely to adopt the chatbot. | Randomised Control trials | None | None | None *Survey results *SUS Score-user experience 10-item Likert scale , MUX score-mobile user experience, PTS score-experience of task difficulty | Other measures *Likert Scales *Evaluated by users | Text | Rule-based NLP engine |
| Trappey A. J. C. et al. | *120 University Students  *Age, Sex, Year of study, Department, Stress level, psychological sensitivity, and Life impact of participants were recorded *Training dataset obtained from the CounselChat website *Fine-tuned the model using BertForSequenceClassification. *Evaluated the test set using Mathews Correlation Coefficient (MCC). | Virtual reality empathy-centric counselling chatbot to provide complementary support for troubled students when counsellors cannot provide immediate support. | *Participants were divided into 2 groups based on the median stress level in the questionnaire.  *Pre-test and post-test questionnaires were compared. | Decreasing average stress level and psychological sensitivity after the experiment. | Quasi-experimental | Classification model for user sentiment/emotion. | Roger's definition of empathy. *Primary level-responding to the client’s explicitly expressed meaning and feelings  *Second level - respond to implied feelings of the person with corresponding emotional words. *Third level - recognizing the client’s confusing and contradictory feelings *Highest level - Guessing suppressed client feelings, from what they are describing. and responding to it directly or indirectly in an acceptable way to the user. | Questionnaire results:  *Stress Level-Decrease in stress levels of users as a measure of effective empathy. *The Psychological Sensitivity (the diversity of emotional effects) and Life Impact (behavioural, physical, cognitive, and social effects) - 5 point Likert scale | *Measured using a 10-point Likert scale.  *Evaluated by users | *Voice interaction  *Multi-turn conversations (verbal or text) | *BERT transformer architecture-Masked Language Model (MLM) and Next Sentence Prediction (NSP) *Automatic Speech recognition model converts speech to text  *Natural Language Understanding (NLU) module converts text into useful information *Dialog state is saved in Tracker and a message is exported to the user, finally the Text-to-Speech model reads the text message to the user in voice. *Sentiment analysis |
| A. Ghandeharioun et al. | *39 participants *Gender and employment were recorded *user ID, baseline scores of the Big Five personality test [40], PANAS (Positive and Negative Affect Scale, short version) [41], and DASS (Depression, Anxiety and Stress Scale) [42]. PANAS quantifies mood and DASS captures depression, anxiety, and stress symptoms.  *Emotionally expressive delivery randomly assigned to each user | Delivery of just-in-time mental Health interventions by emotion-aware chatbot, EMMA. | *Randomly assigned participants to treatment and control groups for the intervention. | EMMA was perceived as likable via self-reports of emotion from users. | Randomised Control trials | *User mood prediction from sensor data - Russel’s two-dimensional model of emotion | None | *None Behavioural metrics *Response latency - the interplay between the emotional intelligence of the bot and intervention engagement *Frequency of Response to Interventions intervention engagement. Survey results. *User Preference- satisfaction and efficacy of the system. | *None *Response latency extracted from the application logs of user clicks in the app *Efficacy evaluated by users | Text | Hybrid engine *Machine Learning model to infer user mood. *Rule-based engine for random response selection from scripted phrases. *Emotion Classification engine-implemented binary classifiers for valence (negative/positive) and arousal (low/high) |
| J. Meng and Y. N. Dai | *278 participants from Midwestern university. *The ones who did not complete survey questions, did not have chat records, failed to follow instructions, and asked irrelevant questions were excluded. *211 final cases meeting inclusion criteria- the average age was 20.4 (SD = 2.28) and 61.6% were females. | A web-based experiment to check if the chatbot’s emotional support was effective in reducing people’s stress and worry. | *Control vs. treatment conditions were conducted within the web-based experiment. | A self-disclosing chatbot without emotional support reduced even less stress than a chatbot not providing any response to participants’ stress. | Randomised Control trials | None | None | *Questionnaire answers-user perception Additionally, *Perceived stress scale- self-reported measures using a 7-point scale (1=strongly disagree) *A single-item measure to measure worry (7-point Likert scale) e.g.- How worried do you feel about this situation? *Scale of Perceived Social Support (MSPSS)- for the measure of Perceived supportiveness of a partner *Neuroticism- A covariate -7-point scale | *A(1=' yes' or 2='no') answer for check questions *Likert scales for the ratings *Evaluated by users. | Facebook Messenger as a pop-up window | Generative Pre-trained (GPT) Transformer engine by OpenAI- developed using a tool called Chatfuel. |
| R. Goel et al. | Dataset: *Trained with the Facebook AI Empathetic Dialogue dataset - with 24,850 Dialgue conversations. Word embeddings(word2vec) were performed with the vector size being 100.  *Model trained using the RMSProp Optimizer. *Automatic evaluation of machine translation using BLEU score. | Empathic CA with attention mechanism to support users express their feelings and anxious thoughts. | *Compared the BLEU score of the model with other similar models | Generates better quality empathetic responses and is better at capturing human feelings and emotions. | Cross-sectional | Capture user emotion using the attention mechanism of the transformer model. | None | None | Not done Automatic evaluation only | Text | *Neural network framework *Seq2seq encoder-decoder architecture that uses the Long Short-term Memory (LSTM)with Word embedding *A modification of the same model with an attention mechanism. |
| A. Adikari et al. | Dataset: *Dataset from Cancer Chat Canada with 120,000 conversations by 320 patients *User dialog dataset from Kaggle (public) -3,000,000 messages (average 5 messages per user) | A conversational agent with emotional state prediction for empathetic patient-centred mental healthcare | Emotion classification accuracy was compared based on the F1 score. | improved mental health and well-being outcomes through natural language processing techniques and artificial intelligence algorithm-based CA | Cross-sectional | *Using an emotion recognition model for patients. -Emotion feature extraction using the model proposed by Plutchik.(anger, fear, sadness, disgust, joy, surprise, trust, anticipation ) -Trained using two-word embedding models word2vec and GloVe. -Markov chains are used to model the emotion state changes. -A 2nd order Markov model used to predict the emotion. *Evaluation done by a group of clinicians and therapists. | None | None Behavioural metrics *Patient behavioural score- for engagement. *Emotion scores- emotion changes over time in a session | Clinicians did the validation of the results | Text | Hybrid engine *NLP engine for emotion recognition-two-word embedding models for emotion expression mining. *rule-based engine for response generation. *Empathic responses are generated using a rule-based emotion message generation model. *Resource recommendations are provided for the patients. |
| B. Inkster et al. | *129 users meeting the inclusion and exclusion criteria. Anonymous voluntary global users of the mobile app, Wysa who self-reported symptoms of depression. | An empathetic, text-based conversational mobile mental well-being app, Wysa, is for users with symptoms of depression. | *Comparison between high users and low users of the app *compare and contrast the  quantitative and qualitative results and help corroborate findings. | The high user’s group had a significantly higher average improvement compared with the low users group. | Quasi-experimental | None The PHQ-9 generated scores -associated with each of the nine Diagnostic and Statistical  Manual of Mental Disorder-e to monitor  improvement or worsening of symptoms of depression | None | None Other - Questionnaire for screening depressive symptoms | Other measures *Evaluated by app users | Text | *Machine learning NLP engine *supervised ML classifier algorithm |
| J. L. Beredo and E. C. Ong | *senior high school and college students between 17 to 20 years old. Dataset: 1.EMPATHETICDIALOGUES Dataset, prompts and utterance features were considered-24,850 conversations grounded on emotional situations 2. Well-being Conversations-local collection of well-being-related conversation logs from a rule-based chatbot. 3. PERMA Lexica-useful in predicting well-being with the use of PERMA scales | An empathetic conversational agent for students to freely talk about their feelings and emotions without feeling invalidated and help them maintain their well-being. | The perplexity score was compared with the Vanila DialoGPT language model. | *VHope were 67% relevant, 78% human-like, and 79% empathic. *Vhope tried deeper conversation with empathy and appropriateness with an uninterested user. *VHope was able to act as a peer or friend *Show compassion when offering advice and comforting responses to its users. | Cross-sectional | *The CA hybrid engine recognises emotion labels and PERMA labels from user input. | None | *Qualitative human evaluation for affect criterion- read and respond to the moods of the user with empathy. *perplexity, metrics for evaluating the model’s language fluency | *Affect criterion/empathy was measured by rating 0 (low) ,100 (high) *Evaluated by 3 experts who studied and practice psychology *Expert evaluation proved that generated responses are 79% empathic. Other measures-performance, humanity | Text | *A hybrid engine *Retrieval-based and generative models for empathetic response generation. *The retrieval model includes emotion detection and Seligman’s PERMA labelling (model with five core elements of psychological well-being, positive emotions, engagement, relationships, meaning, and accomplishment.) *The generative model is the Fine-Tuned Empathetic Responses (FTER) model, which formulates a more empathetic and nonrepetitive response to add variety to the rescripted responses. |
| Rathnayaka P. et al. | *Australian mobile users on google play store (34 eligible individuals across the world)  *user selection criteria; use a smartphone to answer the survey and perform “feelings check-ins” using Bunji app. | A Mood tracking, personalised conversing, remote health monitoring chatbot. | None | Effective in providing support for individuals with mental health issues. | Quasi-experimental | Emotion detection NLP engine-computing an alert score based on the frequency and intensity of words, phrases, and emotions | empathetic engagement means, “making the impression of a credible and trustworthy conversation partner that can hear you out and offer a detached point of view on things” | None *Self-reports for mood scores *Questionnaire for depressive symptom check *metric of mood improvement - this is attributed to recurrent emotional support/empathy | *Measured from a feelings check pre-test to post-test (at least 7 days apart)  *Using pre-trained emotion recognition and sentiment analysis models, a mood score between 0 and 10.- a measure of mood *Evaluated by Bunji users | Text | ML NLP engine *NLU engine and ML *feature extractor (BERT Language model), intent and entity extraction (DIET classifier), and response selection. *Word embeddings |
| R. R. Morris et al. | *Participants included 37,169 individuals who signed up for Koko between mid-August and mid-September of 2016. Corpus from Koko platform: *peer interactions consisted of 72,785 posts and 339,983 responses | A CA with a back-end system to automatically pair archived responses against incoming posts. | Koko users were assigned to a controlled study (interacted with other humans) and a treatment study (with the agent). | Responses created by the agent (79.20%) were deemed acceptable by users. | Randomised Control trials | None | None | None *A user quality rating of the CA responses as good, ok, or bad. | Other measures  *single-item, three-point Likert scale (good, ok, bad) *Evaluated by users | Text | Hybrid engine- Retrieval engine and neural networks retrieval techniques and word embeddings were used to select historical responses that best matched a user’s concerns. |
| A. Ghandeharioun et al. | N=39 participants *7 were females and 32 were males. *17 full-time employees (FTE), 17 interns, and 5 external members or contractors. *Ages ranged between 16 and 49 (M=29.4, SD=7.9). *Depression Anxiety Stress Scales (DASS) scores for mental health and wellbeing status | A conversational bot interface that conducts experience sampling. | *Treatment condition (Emotionally expressive dialog with emojis) vs. control condition (Scripted texts are neutral, not affective) | *Participants reported a higher percentage of positive mood reports when interacting with the empathetic bot. *Extraverts preferred the emotion-aware chatbot significantly more than introverts. | Randomised Control trials | *Self-reported user mood ratings through experience sampling -based on Russel’s two-dimensional model of emotion. | None | None *Russel’s two-dimensional model of emotion - to rate their mood *Survey Answers to questions about agent intelligence and its tone and likability. | * A Likert scale, ranging from 1 (strongly disagree) to 7 (strongly agree) *Evaluated by users | Text | *A rule-based design tree for response selection-The content is selected from the pool of scripted texts. |
| T. Saha et al. | Dataset: *MotiVAte1 dataset- 4k dyadic conversations between the depressed support seekers, | A VA capable of generating empathic and motivational responses in online mental health support | *Compared the performance of the baselines and the proposed model on automated metrics (Perplexity, BLEU, ROGUE-L, Embedding metric) | The VA should be capable of generating empathetic and motivational responses, continuously demonstrating positive sentiment by the VA. | Cross-sectional | None | Empathy or empathic interactions, the ability to feel the emotions and experiences of others. | *Using a framework EPITOME, for identifying expressed empathy for rating responses. | *three human evaluators were recruited to rate the quality of 100 simulated responses average fluency, adaptability and motivational scores of 3.9, 2.63 and 3.82, respectively. *EPITOME consists of 3 mechanisms- -Emotional reactions, Interpretations, and explorations rated 0(not expressing them at all), 1(expressing them to some weak degree), 2(expressing them strongly). Other measures, *Average fluency, adaptability and motivational scores of 3.9, 2.63 and 3.82, respectively. | Text | Transformer based engine *Motivational Response Generator (MRG) uses the pre-trained GPT-2 model from OpenAI. *Empathetic Rewriting Framework (ERF)-understand empathy based on three communication mechanisms: Emotional Reactions, explorations and Interpretations, and transformer-based framework. |
| M. Agnihotri et al. | Dataset: *ScenarioSA , a large-scale conversational dataset with utterance-level affective state labels(Positive, Negative, Neutral). | Topic-driven and Affective Conversational Agent for mental wellbeing. | A comparative analysis of the individual modules of Topic driven affective Conversational agent (TACA) and Topic-Driven Mixture of Empathetic Listeners (TMoEL) | Generated responses achieve significant emotional relevance and are contextually relevant to the conversation topic. | Cross-sectional | None | None | Response ratings | *The emotional relevance of every generated response was rated on a score between 1 to 5, 1 being the lowest.  *Evaluated by three human annotators- male non-native English speakers from a technical university with an average age of 21. Other measures- Contextual Relevance | Text | *Generative Pre-trained Transformer (GPT) |
| K. Rani et al. | None | A chatbot, at the heart of the mental health interaction portal Saarthi. | None | *Quality mental health care from the comfort of their own homes, making it a convenient and accessible solution. | Quasi-experimental | None | None | None | None | Text | ML NLP engine *NLU engine and ML techniques *Term Frequency-Inverse Document- A Word's significance in a document assessed by frequency (N-gram model) *Sentiment analysis |
| L. Alazraki et al. | *N=23 participants, through crowd-working websites Amazon Mechanical Turk and Prolific, were educated at college level or above and their first language was English, age and sex were recorded. *two clinicians Dataset:  * Crowd-sourced the EMPATHETICPERSONAS dataset by  distributing four surveys.  *Using discrete numerical labels from 0 to 2 (where 0 corresponds to a non-empathetic utterance and 2 to a strongly empathetic one). The overall empathy score was computed based on the majority label. *Two annotators who volunteered and have worked in healthcare and are experienced in communicating empathetically with patients. | A CA with human-like personas that users can choose to interact with. | 5 chatbot personas were compared for their results | Achieving a framework with higher empathy, user engagement and usefulness than a simple rule-based framework. | Cross-sectional | Emotion recognition using a language model, RoBERTa | The definition of empathy given by Barrett-Lennard *A first phase- where the listener sympathises and  resonates with what is being expressed by the speaker,  *A second phase in which the listener compassionately responds to  the speaker,  *A third phase where the speaker assimilates  the listener’s response. | Questionnaire results- | *Multiple-choice questions asking the user to evaluate: (a) the chatbot’s ability to display empathy; for each persona *Perceived level of empathy rated from strongly disagree to strongly agree (A 5-point Likert scale) *Two separate clinicians specialised in mental health, also evaluated the chatbot personas Other measures- Fluency function, Novelty function | Text | A hybrid engine * rule-based engine- with deep learning classifier for emotion identification from user text  *retrieval engine for producing novel, fluent and empathetic utterances. |
| M. R. Gundavarapu et al. | Dataset: *A dataset was created using sources like Wikipedia and other relevant Google articles.  *The dataset was updated with frequent conversations with the chatbot. | A chatbot companion for emotional support, without judgement, and with a positive response. | None | Effective in responding to the user with a positive sense to change and instil confidence in them. | Cross-sectional | None | None | None | None | Text | Neural network-based NLP engine *Deep neural network with 3 Dense layers *preprocessing operations on the dataset are: Converting the corpse into lowercase., Tokenization of data, removing noise and stopping words from data, stemming of data, Lemmatisation of data. |
| K. Mishra et al. | Dataset: A novel, high-quality, and large-scale mental health and legal counselling conversational dataset, named MHLCD was created. | A dialogue system for mental health and legal counselling assistance. | *Comparison of the model with baseline models. | Results also support the use of a designed reward function to better facilitate the counselling, politeness and empathy in generated responses. | Cross-sectional | None | None | User rating | *Integer scale of 1-5 (5=High) *Six evaluators rated each dialogue interaction for empathy. *Cross-validated for quality by government-run institutions. | Text | Transformer engine *Three annotators with post-graduate qualifications and significant experience in the related tasks labelled the dataset manually. -counselling strategy, empathy, and politeness labels as per the guidelines provided *Evaluated using automatic and human evaluation metrics. |
