# Supplementary figures and images for: Empathic Conversational Agent Platform Designs and Their Evaluation in the Context of Mental Health: Systematic Review
Source: JMIR Ment Health. 2024 Sep 9;11:e58974. doi: 10.2196/58974 (PMC11420590; doi:10.2196/58974)

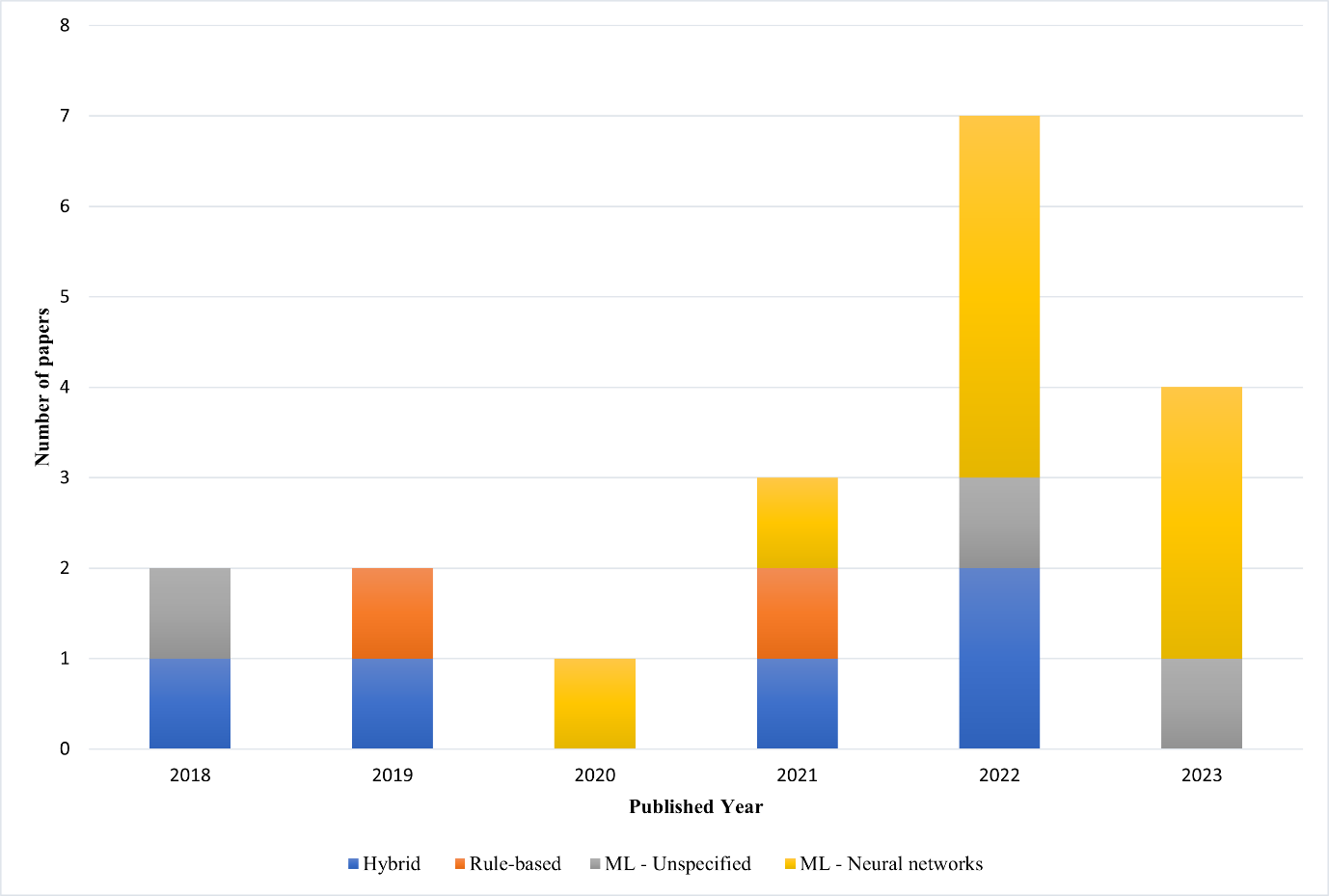


Figure S1- Types of CA Architectures used over the years.

Supplement: Multimedia Appendix 3 [file mental_v11i1e58974_app3.docx]
