# Supplementary material for: Empathic Conversational Agent Platform Designs and Their Evaluation in the Context of Mental Health: Systematic Review
Source: JMIR Ment Health. 2024 Sep 9;11:e58974. doi: 10.2196/58974 (PMC11420590; doi:10.2196/58974)
Supplement: Multimedia Appendix 4 [file mental_v11i1e58974_app4.docx]

| **Type of Architectures** | **Description** | **Proportion** |
| --- | --- | --- |
| **ML engines** | A generic term used to describe any algorithm used to classify input according to several pre-specified categories. ML algorithms must be provided with ground-truth data, against which new input data is compared. | 63% |
| - Neural networks | A broad class of ML models that differentially weight inputs and use feedback for recursive evaluations of error to refine classification accuracy. | 47.25% |
| - Transformer based | A ML algorithm that analyses all parts of a textual input (e.g., all words in multiple sentences) simultaneously or in parallel. This allows the Transformer to propose candidate keywords missing in input, based on contextual cues. Transformers have been used effectively in Natural Language Processing (NLP) and speech processing tasks, including ChatGPT. | 36.85% |
| - Other neural networks |  | 10.4% |
| - Unspecified |  | 15.75% |
| **Hybrid engine** | A ML algorithm that combines two or more model types to improve efficiency and classification accuracy. The combination of a model that clusters keywords into themes with a classification model is one example. | 26% |
| - ML and Rule-based |  | 15.6% |
| - Multiple ML models |  | 10.4% |
| - - Retrieval and Generative engines |  | 5.2% |
| - - Retrieval and Neural network engines |  | 5.2% |
| **Rule-based engine** | An algorithm that applies logical rules (applying Boolean logic) to provide structured responses or actions matched to pre-specified inputs. | 11% |

Table S1: Types of CA Architectures


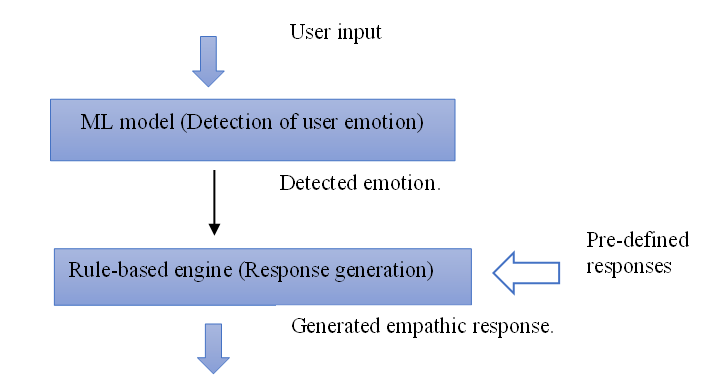

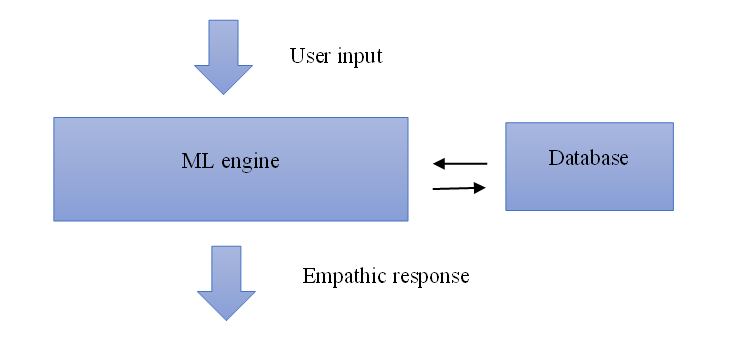


Figure S2: An example of a Hybrid Engine Architecture

Figure S1: An example of a Single Engine Architecture
