# Supplementary material for: Empathic Conversational Agent Platform Designs and Their Evaluation in the Context of Mental Health: Systematic Review
Source: JMIR Ment Health. 2024 Sep 9;11:e58974. doi: 10.2196/58974 (PMC11420590; doi:10.2196/58974)
Supplement: Multimedia Appendix 5 [file mental_v11i1e58974_app5.docx]

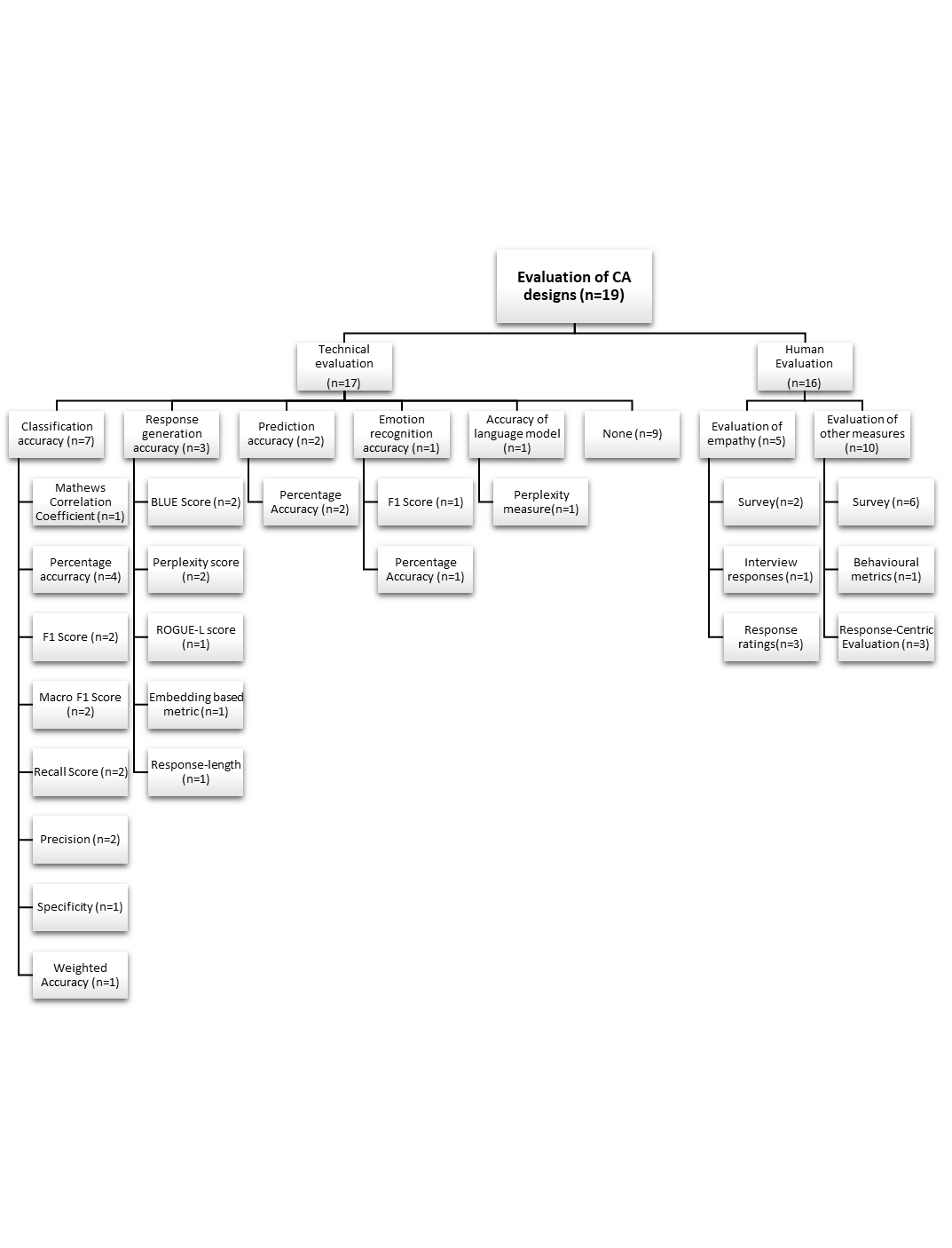


Figure S1: Evaluation of CA designs

Table S1: Other measures for assessing the effectiveness of the CA designs.

| **Author** | **Other measures** | **How was it measured?**  **Who did the evaluation?** | **Effectiveness** |
| --- | --- | --- | --- |
| B. Persons et al. | Survey results *SUS Score-user experiences using a 10-item Likert scale with standardised scores that range between 0 and 100. *MUX score-mobile user experience using a 15-item multifaceted survey *PTE score-experience of task difficulty using a 5-point Likert Scale  * Behavioural intention (BI), perceived ease of use (PEOU), usefulness (PU). | *Likert Scales *Evaluated by users | SUS score  -The laptop group= “good” range (79.58)  -mobile group = “excellent” range (91.67)  -The difference in SUS scores between the two groups was almost significant (p = 0.07).  MUX score  -Both groups were in the above average range (4.33 and 4.44 out of 5)  PTE score  -Both groups were in the above average range (4.28 and 4.39 out of 5) showing little to no difficulty to complete the task.  BI scores  Mobile group - mean of 4.00 on a 5-point scale which is above average.  Laptop group - mean of 2.67 indicating ratings in the lower range of the 5-point scale.  PEOU and PU Score  -Both groups were in the above average range (3.83 and 4.44 for PEOU), 4.28 and 4.83 for PU meaning users found it easy to use and useful. |
| J. L. Beredo and E. C. Ong | Response ratings  *Performance  *Human likeness | *Measured by using a binary scale of 0 (No) -1(Yes) *Evaluated by three experts who studied and practice psychology | Responses were 67% relevant, 78% human-like. |
| Trappey A. J. C. et al. | Survey results:  *[1] Level-Decrease in stress levels of users as a measure of effective empathy. *The Psychological Sensitivity (the diversity of emotional effects) *Life Impact (behavioural, physical, cognitive, and social effects) - 5-point Likert scale | *Measured using a 10-point Likert scale to scale questions *Evaluated by users | Mean stress level of treatment group (TG) decreased from 6.53 to 5.32.  Mean psychological sensitivity of the TG decreased from 1.78 to 1.74.  Mean Life impact of the TG increased from 1.2 to 1.37. |
| A. Ghandeharioun et al. | Behavioural metrics *Response latency - the interplay between the emotional intelligence of the bot and intervention engagement *Frequency of Response to Interventions- for intervention engagement.  Survey results. *User Preference- satisfaction and efficacy of the system | *Response latency extracted from the application logs of user clicks in the app -by author *Efficacy evaluated by users | Average latency of response to interventions = 20.5 minutes which is higher than the control group.  Average frequency of response to intervention = 18.5, higher than the control group.  A statistically significance between the control and treatment conditions were not observed.  Two one-sided t-tests:  t(38) = 5.31, p << 0.0001 and t(38) = −6.33, p << 0.0001 shows agent likability is practically equivalent before and after deploying ML. |
| J. Meng and Y. N. Dai | Survey results *Perceived stress scale- self-reported measures *worry  *Scale of Perceived Social Support (MSPSS)- for the measure of Perceived supportiveness of a partner *Neuroticism  Manipulation check questions *Emotional support and reciprocal disclosure- | *A 7-point (1=strongly disagree) Likert scales for the ratings  *A (1=' Yes' or 2='No') answer for check questions *Evaluated by users. | Perceived stress-  Mean_pre_ = 4.25, SDpre = 0.81,  Mean_post_ =3.90, SDpos t= 0.87.  Worry-  Mean_pre_ =4.94, SDpre = 1.20,  Mean_post_ = 4.54, SDpost = 1.38.  Perceived supportiveness of a partner Mean=3.94, SD=1.25 on a 7-point scale.  Neuroticism  Mean= 4.31, SD =1.25  Emotional support was significantly associated with whether their conversational partners comforted them during the chat,  Chi-squared (1, N=183) =71.72, p < .001, Cramer’s V = 0.63.  Reciprocal disclosure significantly associated with whether their conversational partners self-disclosed during the chat.  Chi-squared (1, N=183) =127.93, p < .001, Cramer’s V = 0.84. |
| B. Inkster et al. | Survey results *A measure of depressive symptoms using the Patient Health Questionnaire (PHQ-9) | *Qualitative feedback using pre-formatted response options *Evaluated by app users | High users  Decrease in mean PHQ-9 scores from 18.92 to 13.07.  Low users  Decrease in mean PHQ-9 scores from 19.86 to 16.33. |
| Rathnayaka P. et al. | Survey results  *Self-reports for mood scores *Survey for depressive symptom check (PHQ-9) *metric of mood improvement - this attribute to recurrent emotional support/empathy | *Measured from feelings -check pre-test to post-test (at least seven days apart)  *Using pre-trained emotion recognition and sentiment analysis models, a mood score between 0 and 10.- a measure of mood *Evaluated by users | Mean mood scores significantly increased from 5.79 to 7.38 post-usage. |
| R. R. Morris et al. | Survey results *A user quality rating for each response | *Single-item, three-point Likert scale (good, ok, bad) *Evaluated by users | 79.20% (2986/3770) of responses from the agent were deemed ok or good.  51.6% (227/440) rated the agent responses as ‘good’ compared to 60.6% (510/842) ratings of peer responses as ‘good’. |
| A. Ghandeharioun et al. | Survey results -*User preference via questions about agent intelligence and its tone and likability.  *User satisfaction ratings-  -Big Five Personality Traits  -Positive and Negative Affect Schedule | * A 7-point Likert scale used to provide measurements for user preference  *5-point Likert scale for user satisfaction *Evaluated by users | Agent likability scores for extroverts-  Mean=5.17, SD=0.91  Agent likability scores for introverts-  Mean=4.43, SD=0.55  Percentage of positive emotions for Emotion-Aware (Mean=80.55, SD=3.65) and Control (Mean=69.08, SD=4.16) conditions has a significant difference.  t(37)=2.74, p = .009 |
| T. Saha et al. | Response ratings  *Average fluency - grammatically correct responses *Adaptability - responses aligned with the context *motivational scores - positively-oriented responses imparting hope and motivation | *Testing on a scale of 1 (worst) to 5 (best) *Three human evaluators were recruited to rate the quality of 100 simulated responses. | Highest average fluency, adaptability and motivational scores of 3.9, 2.63 and 3.82, respectively. |
| M. Agnihotri et al. | Response ratings *Contextual Relevance of responses | *Contextual relevance - rated on a score between 1 and 5, 1 being the lowest.  *Evaluated by three human annotators- male non-native English speakers from a technical university with an average Age of 21. | When empathetic response generator is used,  Contextual relevance= 2.87  When a topic classifier is added,  Contextual relevance= 2.11 |
| L. Alazraki et al. | Survey results  *Usefulness - the platform was useful or not?  *Preference on chatbot persona *Engagement | *Used 5-point scale from strongly agree to strongly disagree for ratings. *Two separate clinicians specialised in mental health also evaluated the chatbot personas | Usefulness-  75% agreed 17% strongly agreed.  8% choosing ‘Neither agree nor disagree’.  Preference-  60% preferred personas Robert and Arman.  Engagement-  63% agreed that Kai’s conversations were engaging and a further 6% strongly agreed. |

Table S2: Accuracy of CA designs

| **Author** | **Type of CA Architecture** | **Accuracy assessed** | **Results** |
| --- | --- | --- | --- |
| Trappey A. J. C. et al. | ML-based (Transformer-based) | Classification performance | MCC = 0.8570. |
| A. Ghandeharioun et al. | Hybrid | *Classification accuracy *Personalised regression for prediction | Classification accuracy- 80.4% Prediction accuracy - 82.2% |
| R. Goel et al. | ML-based (Other Neural networks) | Accuracy of the response generation | BLEU score of 0.126 |
| A. Adikari et al. | Hybrid | *Classification accuracy  *Emotion prediction accuracy | Classification - F1 score of 0.87. Prediction -  79% -correctly predict the next emotion as positive or negative 63%-correct emotion out of all emotions was predicted |
| B. Inkster et al. | ML-based (unspecified) | Classify objections in real time | Accuracy- 99.2% Specificity- 99.7% Precision- 74.7% Recall- 62.1% |
| J. L. Beredo and E. C. Ong | Hybrid | Performance of the language model | PERPLEXITY MEASURE 9.977 |
| T. Saha et al. | ML-based (Transformer-based) | Performance of response generator | BLEU-1 score-0.161 perplexity- 50.90 ROUGE-L score- 0.124 embedding based metrics: Average - 0.733 Extrema- 0.377 Greedy- 0.478 |
| M. Agnihotri et al. | ML-based (Transformer-based) | Performance of the topic classifier | Accuracy = 95%  Precision = 0.954 Recall = 0.947 F-1 score= 0.95 |
| L. Alazraki et al. | Hybrid | *Emotion recognition *Classification for empathy function | Emotion recognition-  accuracy 94.96% F1 score 95.10%  Classification accuracy- accuracy of 80.18% F1 score of 80.66% |
| K. Mishra et al. | ML-based (Transformer-based) | *Counselling strategy, politeness and empathy classification  *Quality of responses | Classification accuracy of empathy- Weighted Accuracy - 0.977 Macro F1 - 0.972 Response quality- Perplexity score - 1.91 response length- 18.71 |
