# Supplementary material for: Empathic Conversational Agent Platform Designs and Their Evaluation in the Context of Mental Health: Systematic Review
Source: JMIR Ment Health. 2024 Sep 9;11:e58974. doi: 10.2196/58974 (PMC11420590; doi:10.2196/58974)
Supplement: Multimedia Appendix 6 [file mental_v11i1e58974_app6.docx]

Dictionary

| Term | Explanation |
| --- | --- |
| BERT | Stands for ‘Bidirectional Encoder Representations from Transformers’. It is a groundbreaking language model built on transformer architectures, with the ability to read text in both directions simultaneously as opposed to other traditional language models. This was created by researchers at Google. |
| RoBERTa | Stands for ‘Robustly optimised BERT approach’. It is a modification of the BERT model with changes in parameters, such as the learning rate or choice of optimiser, which specifies details of the learning process. |
| SBERT | Stands for ‘Sentence-BERT’. It is a modification to the BERT model to be more suitable for sentence-level comparisons. |
| GPT-2 | Stands for ‘Generative Pretrained Transformer 2’. It is an advanced language model developed by OpenAI for a transformer architecture. It has capabilities like text generation, text summarisation, answering questions and translations. |
| Hybrid response generation | A method used in CA designs where multiple technologies or methods are used to generate replies to user input. |
| Word embeddings | It is how words are represented especially in the form of a vector for effective NLP. |
| Experience sampling | It is a method for collecting data about individuals' experiences in real time. |
| Topic-driven responses | It is when a CA replies to the user based on the topic of conversation with more relevant and meaningful content. |
| Dense layers | This refers to the fully connected nature of the layers in neural network models. |
| Generative model | It is an ML technique that produces unique, contextually relevant replies that are not limited to the CA's initial programming. |
| Retrieval engine | It is a type of ML architecture designed to access information through a database or the internet to be shared with users to match their inquiries. |
| Corpus | A large, structured set of texts is used for analysis mostly in NLP. |
| Valence | Psychology refers to valence as the intrinsic attractiveness or aversiveness of an event, object, or situation. |
| Arousal | A state of feeling energized or alert. |
