# Supplementary material for: Empathic Conversational Agent Platform Designs and Their Evaluation in the Context of Mental Health: Systematic Review
Source: JMIR Ment Health. 2024 Sep 9;11:e58974. doi: 10.2196/58974 (PMC11420590; doi:10.2196/58974)
Supplement: Multimedia Appendix 7 [file mental_v11i1e58974_app7.docx]

Table S1 - Risk of Bias and Quality Assessment

|  |  | **ROB-2** | **ROBINS-I** | **JBI Appraisal Tool** | |
| --- | --- | --- | --- | --- | --- |
| Author | Study Design | Level of Bias | Level of Bias | Average Score - Based on Design and Implementation | Average Score - Based Only on Design |
| Q. Jiang et al. | Qualitative | Not applicable | Not applicable | 0.80 | 0.80 |
| R. Goel et al. | Cross-sectional | Not applicable | Not applicable | 0.25 | 0.13 |
| L. Brocki et al. | Cross-sectional | Not applicable | Not applicable | 0.25 | - |
| A. Adikari et al. | Cross-sectional | Not applicable | Moderate | 0.63 | - |
| J. L. Beredo and E. C. Ong | Cross-sectional | Not applicable | Serious | 0.75 | - |
| T. Saha et al. | Cross-sectional | Not applicable | Moderate | 0.63 | 0.13 |
| M. Agnihotri et al. | Cross-sectional | Not applicable | Moderate | 0.63 | 0.13 |
| L. Alazraki et al. | Cross-sectional | Not applicable | Moderate | 0.75 | 0.13 |
| M.R.Gundavarapu et al. | Cross-sectional | Not applicable | Not applicable | - | - |
| K. Mishra et al. | Cross-sectional | Not applicable | Moderate | 0.38 | - |
| K. Rani et al. | Quasi-experimental | Not applicable | Not applicable | 0.89 | - |
| B. Inkster et al. | Quasi-experimental | Not applicable | Not applicable | 1.00 | 0.44 |
| Rathnayaka P. et al. | Quasi-experimental | Not applicable | Not applicable | 0.89 | 0.44 |
| Trappey A. J. C. et al. | Quasi-experimental | Not applicable | Not applicable | 0.78 | 0.56 |
| B. Persons et al. | Randomised Control trials | Low | Not applicable | 0.69 | 0.54 |
| A.Ghandeharioun et al. | Randomised Control trials | Low | Not applicable | 0.77 | 0.38 |
| J. Meng and Y. N. Dai | Randomised Control trials | Low | Not applicable | 0.69 | 0.54 |
| R. R. Morris et al. | Randomised Control trials | Low | Not applicable | 0.46 | 0.46 |
| A.Ghandeharioun et al. | Randomised Control trials | Low | Not applicable | 0.69 | 0.62 |
